# Cost-utility of ferric carboxymaltose (Ferinject®) for iron-deficiency anemia patients with chronic heart failure in South Korea

**DOI:** 10.1186/1478-7547-12-19

**Published:** 2014-09-10

**Authors:** Eun-A Lim, Hyun-Soon Sohn, Haeyoung Lee, Sang-Eun Choi

**Affiliations:** 1 College of Pharmacy, Korea University Sejong Campus 2511 Sejong-ro, Sejong City 339-770, Korea; 2 College of Pharmacy, Ajou University, 206 Worldcup-ro, Yeongtong-gu, Suwon-si, Gyeonggi-do 443-749, Korea

**Keywords:** Cost-effectiveness, Cost utility, Chronic heart failure, Iron-deficiency anemia, New York heart association (NYHA) functional class

## Abstract

**Background:**

Iron-deficiency anemia (IDA) is prevalent in patients with advanced chronic heart failure (CHF). It affects the patients’ overall physical condition and is suggested as a strong outcome predictor in CHF. Recent clinical trials suggested that intravenous iron supplementation improves CHF functional status and quality of life. The aim of this study was to assess the cost-effectiveness of ferric carboxymaltose(FCM) in CHF patients with IDA.

**Methods:**

Ferric carboxymaltose, an intravenous iron preparation, was compared with placebo. The target population comprised CHF patients with IDA in hospital and outpatient care settings. We conducted this study from the Korean healthcare payers’ perspective with a time horizon of 24 weeks. One clinical trial provided the clinical outcomes of ferric carboxymaltose therapy. The improvement rates of the New York Heart Association (NYHA) functional class in the placebo and ferric carboxymaltose groups were used to estimate effectiveness in the base-case model. We also conducted a scenario 2 analysis using quality of life investigated in the clinical trial. A panel survey was conducted to obtain the ratio of healthcare resource use based on NYHA class in Korea. Cost-effectiveness was expressed as incremental cost (US dollars) per quality-adjusted life-year (QALY) gained.

**Results:**

In the base-case analysis, the incremental cost-effectiveness ratio (ICER) of ferric carboxymaltose compared with placebo was $22,192 (₩25,010,451) per QALY gained. The sensitivity analysis showed robust results, with the ICERs of ferric carboxymaltose ranging from $5,156 to $29,796 per QALY gained. In the scenario 2 analysis, ICER decreased to $12,598 (₩14,198,501) per QALY gained.

**Conclusions:**

Iron repletion with ferric carboxymaltose for IDA in CHF patients was cost-effective compared with placebo.

## Background

Anemia is common in patients with chronic heart failure (CHF), having a prevalence rate of approximately 30% [1]. However, reported prevalence rates vary between 4% and 70%, depending on the population studied. In Korea, the reported prevalence was 36% [2]. The prevalence of anemia was reported to increase dramatically with an increase in CHF severity [3]. The most commonly encountered type of anemia in CHF is normocytic anemia, which is most frequent in chronic disease, but iron-deficiency anemia (IDA) which is microcytic anemia was found in 73% of patients with advanced-CHF [4].

Anemia in CHF patients is caused by various factors. The main pathological mechanism of anemia is either reduced response to erythrocyte production in the kidney due to hypoxia [3] or diluted blood due to volume repletion [5,6]. Anemia may be a predictor of mortality in CHF patients [7]. Previous studies reported anemia as an independent negative prognostic factor of mortality [2,8,9]. According to the European Society of Cardiology (ESC), anemia is associated with various symptoms, worsened functional status, increased risk for CHF, increased hospitalization, and decreased survival; thus, it needs to be diagnosed and managed [10]. However, guidelines for the management of anemia in CHF patients have not been established. The ESC recommends correction of anemia only after identifying the cause. A transfusion is not recommended, but erythropoietin-stimulating agent (ESA) therapy with iron may be considered [10].

IDA, one type of anemia, also has a negative impact on overall physical condition because it reduces aerobic activity via its negative effect on erythropoiesis, oxidative metabolism, and cellular immune mechanisms [11]. Compared with anemia, IDA was suggested as a stronger and independent outcome predictor in CHF [12]. However, IDA management in CHF patients still tends to be easily ignored [4,13,14]. Recent randomized controlled trials suggested that intravenous (IV) iron supplementation improves CHF functional status and quality of life (QoL) [15-18]. Recently, interest in iron therapy has increased owing to the various negative effects of blood transfusion or ESA therapy.

The aim of this study was to analyze, from the Korean healthcare payers’ perspective, the cost-effectiveness of ferric carboxymaltose (FCM), an IV iron preparation, for the treatment of anemia in CHF patients in comparison with no treatment.

## Methods

The cost-effectiveness of FCM (Ferinject®, Vifor Pharma) was analyzed in comparison with placebo in CHF patients with IDA. Cost-effectiveness analysis (CEA) is used in outcomes research as a method to evaluate both the cost and effectiveness of a treatment for estimation of its relative value in the treatment group compared with the control group.

CHF patients with IDA in inpatient and outpatient care settings were included as the target population. The target population was defined according to the codes of the *Korean Standard Classification of Diseases, 5th Edition* (*KCD-5*), which are based on the codes of the *International Classification of Diseases, 10th Revision (ICD-10).* The *KCD-5* codes for CHF were I50, I500, I501, and I509, and the ICD-10 codes for IDA were D50, D500, D501, D508, and D509.

For comparator selection, we initially considered both active comparator with other iron preparations and no treatment which is important because IDA correction has been easily ignored in the management of CHF. After a systematic literature search, we had to choose only placebo as a comparator.

We identified one clinical trial, the Ferinject Assessment in Patients with Iron Deficiency and Chronic Heart Failure (FAIR-HF) study, performed by Anker et al. [16], in which the efficacy of IV iron therapy with FCM was evaluated in CHF patients with IDA in comparison with placebo. We used the change in the New York Heart Association (NYHA) functional class from baseline to a 24-week follow-up period as the clinical outcome for both the placebo and FCM groups.

**Figure 1:**
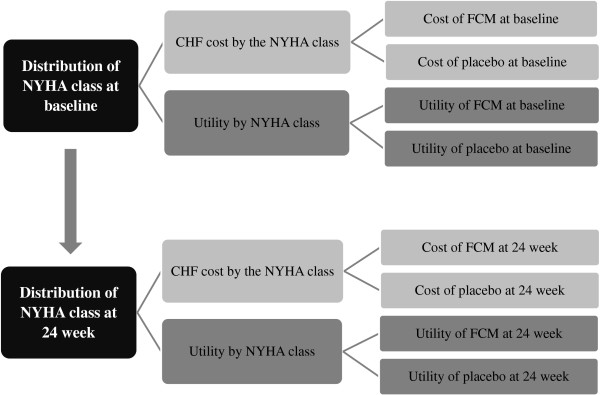
**Model diagram.** A cost-effectiveness model was constructed according to the changes in NYHA class from baseline to 24 weeks in the placebo and FCM groups. The key assumptions of the model were as follows: 1) the effect of the intervention was immediate and lasted throughout 24 weeks in the placebo and FCM groups, and 2) no difference in safety was observed between the 2 groups.

The time horizon was 24 weeks in accordance with the follow-up period in the clinical study. A cost-effectiveness model was constructed based on the change in the NYHA class from baseline to 24 weeks in the placebo and FCM groups (Figure 1).

In the model, utility gain was defined as the result of increased utility based on improvement in NYHA class. We systemically searched PubMed-Medline and the cost-effectiveness analysis (CEA) registry provided by the Tufts Medical Center to determine the utility weights at the NYHA class I, II, III, and IV health statuses. Meanwhile, FAIR-HF investigators studied QoL and converted the QoL into utilities [19], unlike the utilities due to change in the NYHA classes in our study. Thus, we also evaluated cost-effectiveness with the result in a scenario 2 analysis.

The quality-adjusted life-year (QALY) gain was estimated under the assumption that the effect of the intervention was immediate and lasted throughout the 24 weeks in both study groups. According to clinical experts’ opinion, when oral iron is administered to IDA patients, QoL is improved within 12–24 hours via the replenishment of iron enzyme, erythrocyte level is increased within 36–48 hours, and the maximum reticulocyte level is achieved within 5–7 days. Because FCM was intravenously administered, we assumed that the effect would be immediate. In addition, no statistically significant difference in adverse events was observed between the 2 groups in the study of Anker et al. [16]. Therefore, we assumed no difference in adverse events between the 2 groups and did not consider it in the model. The incremental cost-effectiveness ratio (ICER; i.e., incremental cost [US dollars] per QALY gained) was calculated by dividing the incremental cost by the incremental utility between the 2 groups according to the following formula:

$$\begin{array}{c} \mathrm{ICER}=\frac{\left(\mathrm{CD}\;\mathrm{from}\;\mathrm{baseline}\;\mathrm{to}\;24\;\mathrm{weeks}\;\mathrm{in}\;\mathrm{FCM}\right)-\left(\mathrm{CD}\;\mathrm{from}\;\mathrm{baseline}\;\mathrm{to}\;24\;\mathrm{weeks}\;\mathrm{in}\;\mathrm{placebo}\right)}{\left(\mathrm{UD}\;\mathrm{from}\;\mathrm{baseline}\;\mathrm{to}\;24\;\mathrm{weeks}\;\mathrm{in}\;\mathrm{FCM}\right)-\left(\mathrm{UD}\;\mathrm{from}\;\mathrm{baseline}\;\mathrm{to}\;24\;\mathrm{weeks}\;\mathrm{in}\;\mathrm{placebo}\right)}\\ \left(\mathrm{UD}=\mathrm{utility}\;\mathrm{difference},\mathrm{CD}=\mathrm{cost}\;\mathrm{difference}\right) \end{array}$$

From the Korean healthcare system perspective, treatment costs included the direct medical costs added to the non-reimbursable medical costs. The direct medical costs included: 1) cost of IDA treatment with FCM (for the FCM group only) and 2) the average medical cost per NYHA class in the CHF patients with IDA, excluding the total cost of IV iron treatment, which was composed of IV iron drug and administration costs. The cost of IDA treatment with FCM was calculated using the microcosting approach. The average medical cost for CHF per NYHA class was estimated using the macrocosting approach with aggregated healthcare insurance claims data. To assess the average total medical cost (average medical cost including total cost of IV iron treatment) for the CHF patients with IDA, a regression model was constructed to adjust for age, sex, insurance type, and illness severity between the IDA and non-IDA patients, using sampled data from the 2009 National Health Insurance Patient Data. The adjusted average total medical cost for the CHF patients with IDA from the regression model was US $3,161, from which the average total cost of IV iron treatment of US $59 was subtracted for the input of the average medical cost in the cost-effectiveness model (Table 1).

**Table 1:** Estimation of the annual medical cost for the CHF patients according to NYHA class

	**NYHA class**				
	**I**	**II**	**III**	**IV**	**Source**
Average medical cost per CHF patient with IDA (1 year) (US$)					
Adjusted total medical cost: A	3,161				NHI claims data
Total cost of IV iron treatment*: B	59				NHI claims data
Adjusted total medical cost (excluding cost of IV iron treatment): C	3,102				(A-B)
Average healthcare resource use per CHF patient according to NYHA class (1 year)					
Days of hospital stay: D1	2.11	5.74	11.61	24.68	Clinical expert survey
Numbers of outpatient visit: D2	3.76	4.92	7	11.32	Clinical expert survey
Average unit cost of healthcare resource use per CHF patient (US$)					
Inpatient cost per hospital day: E1	81				NHISY
Outpatient cost per outpatient visit: E2	80				NHISY
Cost and cost ratio of CHF treatment according to NYHA class based on healthcare resource use					
CHF treatment cost (US$)	472	859	1,503	2,909	(D1×E1)+(D2×E2)
Cost ratio	0.082	0.150	0.262	0.507	
Adjusted annual CHF medical cost per patient (excluding cost of IV iron treatment) according to NYHA class	1,019	1,857	3,246	6,285	C × cost ratio/0.25†

*CHF*, chronic heart failure; *IDA*, iron-deficiency anemia; *IV*, intravenous; *NYHA*, New York Heart Association; *NHI*, national health insurance; *NHISY*, national health insurance statistical yearbook.

*Includes drug cost and administration cost of IV iron.

^†^Because the adjusted medical cost excluding cost of IV iron treatment (C) is the average cost of patients with NYHA classes I, II, III, and IV, the cost ratio was divided by 0.25 to calculate the CHF medical cost according to NYHA class.

However, because the NHI claims data only provided the total CHF medical cost and did not provide the cost according to NYHA class, we took several steps to determine the cost ratio of the patients with NYHA classes I, II, III, and IV. First, we surveyed clinical specialists working in hospitals and clinics. Because physicians were not aware of CHF treatment costs according to NYHA class, we instead asked questions about hospital days and outpatient visits according to NYHA class. The average number of hospital days of the CHF patients with NYHA classes I, II, III, and IV was 2.1, 5.7, 11.6, and 24.7 days per year, respectively, and the average number of outpatient visits was 3.8, 4.9, 7.0, and 11.3, respectively. Second, for the CHF patients, we obtained the unit costs per healthcare resource used from the 2009 National Health Insurance Statistical Yearbook (NHISY): the average inpatient cost per hospital day and the average outpatient cost per outpatient visit [20]. Third, we determined the cost ratio of CHF treatment according to NYHA class based on healthcare resource use. The cost of healthcare resource use in CHF patients was calculated as the sum of multiplying inpatient cost per hospital day by annual hospital days and multiplying outpatient cost per outpatient by annual outpatient visits. The CHF treatment cost ratios for the patients with NYHA classes I, II, III, and IV were 0.082, 0.150, 0.262, and 0.507, respectively.

Finally, the adjusted average annual medical cost by NYHA class in CHF patients with IDA (excluding the total IV iron treatment cost) was calculated by multiplying the adjusted average medical cost and the cost ratio according to NYHA class (Table 1).

All the costs were calculated based on 2012 data, applying the consumer price index, and reported in US dollars (1 US dollar = Kor ₩1,127). Sensitivity analyses were performed to identify uncertainties of main variables by using Microsoft Excel 2010.

This study did not require Institutional Review Board (IRB) review or approval since the data were obtained from the existing studies and any coded private information were not collected.

### Input data

#### Clinical outcome

In the FAIR-HF study [16], the patient distribution according to NYHA functional class at baseline was similar between the FCM and placebo groups as follows: FCM group (n = 304), 17.4% in class II and 82.6% in class III; placebo group (n = 155), 18.7% in class II and 81.3% in class III. After 24 weeks, the proportion of patients with NYHA classes I, II, III, and IV were respectively 6%, 41%, 50%, and 1% in the FCM group, and 1%, 29%, 65%, and 3% in the placebo group. The FCM group showed a significant improvement in NYHA functional class (p < 0.001). Five deaths (2%) were reported in the FCM group; and four deaths (3%) in the placebo group (Table 2).

**Table 2:** Input values in the base case analysis

	**FCM**	**Placebo**	**Source**
Proportion of patients according to NYHA class at baseline			
NYHA class II	0.17	0.19	Anker 2009 [16]
NYHA class III	0.83	0.81	Anker 2009 [16]
Proportion of patients according to NYHA class at 24 weeks			
NYHA class I	0.06	0.01	Anker 2009 [16]
NYHA class II	0.41	0.29	Anker 2009 [16]
NYHA class III	0.5	0.65	Anker 2009 [16]
NYHA class IV	0.01	0.03	Anker 2009 [16]
Death	0.02	0.03	Anker 2009 [16]
Total medication cost (IDA treatment cost)	US $645	0	
FCM price (500 mg vial)	US $160	0	Suggested price
Dose	1,000 mg	0	KNF
No. of infusions	2	0	Gutzwiller 2012 [19]
Normal saline price (200 mL)	US $1.8	0	HIRA weighted price
Infusion fee	US $1.1	0	NHI fee schedule
Adjusted CHF medical cost by NYHA class excluding cost of IV iron treatment (6 months)			
NYHA class I	US $510	US $510	See Table 1
NYHA class II	US $928	US $928	See Table 1
NYHA class III	US $1,623	US $1,623	See Table 1
NYHA class IV	US $3,142	US $3,142	See Table 1
Utility weight according to NYHA class (1 year)			
NYHA class I	0.93	0.93	Fox 2007 [21]
NYHA class II	0.78	0.78	Fox 2007 [21]
NYHA class III	0.61	0.61	Fox 2007 [21]
NYHA class IV	0.44	0.44	Fox 2007 [21]
Utility difference from the FAIR-HF study	0.037 (scenario 2)	Gutzwiller 2012 [19]	

*FCM*, ferric carboxymaltose; *IDA*, iron-deficiency anemia; *KNF*, Korean National Formulary; *NYHA*, New York Heart Association; *NHI*, national health insurance; *No*, Number; *IV*, intravenous.

#### Costs

To calculate the IDA treatment cost, we assumed that 2 doses of FCM were administered and that each dose contained 2 vials of 500 mg FCM diluted in 200 mL normal saline. The FCM dose was based on the average dose (1851.33 mg) in the FAIR-HF study [16] and in an economic analysis study conducted by the FAIR-HF study group [19]. The administration method was based on the instruction described by the Korean National Formulary. The medication and administration costs of FCM were included as the IDA treatment cost, which was $645 for 24 weeks.

The 24-week average medical costs excluding the total cost of IV iron treatment for CHF patients with IDA who had NYHA classes I, II, III, and IV were $510, $928, $1623, and $3142, respectively (Table 2).

#### Utilities

Four primary studies [22-25] that reported utilities based on NYHA class were found in the systematic literature review via the CEA registry and Medline (Table 3). Among them, the study by Göhler et al. [25] was excluded because it reported utilities in CHF patients without comorbidity, which were irrelevant to our study population. Utilities from a pharmaco-economic study by Fox et al. [21] were used in the base-case analysis because these utilities were surveyed in the general population. Fox et al. used results from the study of Kirsch and McGuire [22] as the utilities for NYHA classes I and II and used United Kingdom general population-based utilities [24] derived from the results on health-related QoL in the Cardiac Resynchronization in Heart Failure (CARE-HF) trial [26] as the utilities for NYHA classes III and IV.

**Table 3:** Utility weights according to NYHA subclass obtained from the systematic review

**Source**	**Respondents**	**Assessment method**	**Utilities by NYHA subclass**				**Remarks**
			**I**	**II**	**III**	**IV**	
Fox 2007 [21]	Not primary utility study^†^	-	0.93	0.78	0.61	0.44	Base case
Kirsch and McGuire 2000 [22]	General population, UK	TTO with EQ5D	0.934	0.782	0.553	0.371	2-year TTO
			0.930	0.765	0.509	0.284	10-year TTO
			0.932	0.774	0.531	0.328	Mean, sensitivity analysis
Lewis 2001 [23]	Advanced CHF (EF < 40) patients, USA	TTO, SG, MLWHF and VAS	0.97	0.80	0.65	0.30	Sensitivity analysis
Calvert 2005 [24]	Patients requiring CRT (NYHA classes III and IV) from the CARE-HF trial, multi-country	EQ-5D and MLWHF	-	-	0.61	0.44	Sensitivity analysis
Göhler 2009 [25]	CHF patients in post AMI from the EPHESUS trial, multi-country	EQ-5D	0.9	0.84	0.74	0.6	Utility in the absence of further comorbidities^‡^

*AMI*, acute myocardial infarction; *CARE-HF*, Cardiac Resynchronization in Heart Failure; *CHF*, chronic heart failure; *CRT*, cardiac resynchronization therapy; *EF*, ejection fraction; *EPHESUS*, Eplerenone Post-acute Myocardial Infarction Heart Failure Efficacy and Survival Study; *EQ-5D*, EuroQol-5 dimension; *MLWHF*, Minnesota Living with Heart Failure questionnaire; *TTO*, time trade-off.

^†^The results from the study of Kirsch and McGuire (2000) [21] were used as utilities for NYHA classes I and II, and the results from the study of Calvert et al. (2005) [23] were used as utilities for NYHA classes III and IV.

^‡^This result is not valid because our study population was defined as CHF patients with iron deficiency anemia.

As mentioned earlier, the utility difference between two groups converted from QoL according to the FAIR-HF study investigators was 0.037 [19], which was used for the scenario 2 analysis.

#### Sensitivity analysis

Sensitivity analyses were performed to identify the uncertainty for the CHF treatment cost ratio according to NYHA class obtained from a previous study [27], utility weights obtained from selected studies through the systematic review [22-24], FCM drug price (±15%), total number of FCM vials used (±1 vial), CHF medical cost (±25%), and utilities according to NYHA class (±10%), onset of effect on the sixth day, and distribution of CHF patients at baseline in the expert survey (58.3% and 41.7% for classes II and III, respectively; Table 4). Moreover, sensitivity analyses in the scenario 2 model were conducted for all the input variations, except for utility weight variations. The utility gain in the scenario 2 model did not change according to the different utilities obtained from different sources because the utility gain was directly obtained from the QoL result investigated by the FAIR-HF study group (Table 5).

**Table 4:** Results of the base-case and one-way sensitivity analyses (24 weeks)

**Variables**	**Input values**	**Incremental costs** ** (US $)**	**Incremental ** **QALYs**	**ICER (US $ per QALY gained)**
Base case		466	0.0210	22,192 (₩25,010,451/QALY)
Cost ratio from Berry 2001 [27]		483	0.0210	23,013
NYHA class I	0.049			
NYHA class II	0.049			
NYHA class III	0.120			
NYHA class IV	0.831			
Utilities from the mean of 2 studies based on advanced CHF patients [23,24]		466	0.0224	20,805
NYHA class I	0.97			
NYHA class II	0.8			
NYHA class III	0.63			
NYHA class IV	0.37			
Utilities from the mean of 3 principal utility studies [22-24]^†^		466	0.0239	19,508
NYHA class I	0.95			
NYHA class II	0.79			
NYHA class III	0.60			
NYHA class IV	0.36			
FCM price ±15% (US $)	136-184	370-562	0.0210	17,630-26,754
Total number of FCM vials ± 1 vial	3-5	306-626	0.0210	14,588-29,796
CHF medical cost ± 25%	US$1,185–1,975			20,028-24,356^∮^
Utilities according to NYHA class (± 10%)				
NYHA class I: ± 10%	0.84-1^‡^			20,485-24,954*
NYHA class II: ± 10%	0.70-0.86			17,797-29,469*
NYHA class III: ± 10%	0.55-0.67			17,945-29,073
NYHA class IV: ± 10%	0.40-0.48			21,737-22,667
Effect-onset time	sixth day	466	0.0204	22,873
Baseline CHF patient distribution from the expert survey		191	0.0547	3,496
NYHA class II	58.3%			
NYHA class III	41.7%			

(US $1 = Kor ₩1,127).

*CHF*, chronic heart failure; *FMC*, ferric carboxymaltose; *ICER*, incremental cost-effectiveness ratio; *NYHA*, New York Heart Association.

^†^The mean of 2 results from the study of Kirsch and McGuire (2000) [21] was used.

^‡^A value of 1 was applied instead of 1.023 because utility weight cannot be more than 1, although the utility weight was 1.023 in NYHA class ± 10%.

^∮^As the CHF medical cost increased, the ICER decreased.

*As the utility weight increased, the ICER decreased.

**Table 5:** Results of the scenario 2 and one-way sensitivity analyses (24 weeks)

**Variables**	**Input values**	**Incremental costs (US $)**	**Incremental QALY**^**†**^	**ICER (US $ per QALY gained)**
Base case		466	0.0370	12,598 (₩14,198,501/QALY)
Cost ratio from Berry 2001 [27]		483	0.0370	13,065
NYHA class I	0.049			
NYHA class II	0.049			
NYHA class III	0.120			
NYHA class IV	0.831			
FCM price ± 15% (US $)	136-184	370-562	0.0370	10,009-15,188
Total number of FCM vials ± 1 vial	3-5	306-626	0.0370	8,282-16,915
CHF medical cost ± 25% (US $)	1,185-1,975	421-512	0.0370	11370-13,827^‡^
Effect-onset time	6th day	466	0.0370	12,598
Baseline CHF patient distribution from the expert survey		191	0.0370	5,165
NYHA class II	58.3%			
NYHA class III	41.7%			

(US $1 = Kor ₩1,127).

CHF, chronic heart failure; FMC, ferric carboxymaltose; ICER, incremental cost-effectiveness ratio; NYHA, New York Heart Association.

^**†**^Utility gains in scenario 2 were not changed according to variation of inputs because the utilities were directly obtained from the quality-of-life results at the follow-up periods in the FAIR-HF study.

^‡^As the CHF medical cost increased, the ICER decreased.

### Cost-effectiveness results

#### Base-case analysis

In the base-case analysis, with the assumption that 1000 mg FCM was infusioned twice during a 24-week period, the incremental cost of FCM over placebo was $466, incremental QALY was 0.021, and ICER was $22,192 (₩25,010,451) per QALY gained (Table 4). This means that for IV iron therapy in the CHF patients with IDA, an additional $22,192 was spent to obtain 1 QALY (1 life-year gained in perfect health) compared with that in the patients with no treatment.

#### Sensitivity analysis in the base-case model

From a sensitivity analysis which applied the cost ratio according to NYHA class obtained from the study of Berry et al. [27], the ICER of FCM over placebo was $23,013 (₩25,935,651) per QALY gained, an increase of approximately 4% compared with the base-case result (Table 4). When different utility weights obtained from the systematic literature research were applied to the model, the ICER decreased by approximately 6% under the utilities of the mean of two studies for advanced-stage CHF patients [23,24] and decreased by approximately 12% under the utilities from the mean of three principal utilities [22-24]. By changing the number of FCM vials and FCM prices, which affect FCM total cost, the ICER ranged from $17,630 to $26,754 (₩19,869,010 to ₩30,151,758) and from $14,588 to $29,796 (₩16,440,676 to ₩33,580,092) per QALY gained, respectively. As the average CHF medical cost and utility weights of NYHA classes I and IV increased, the ICER decreased. The model was not sensitive to the average medical cost of CHF. The utility change in NYHA classes II and III affected the ICER more than the utility change in NYHA classes I and IV. When no effect occurred in the first 5 days, the ICER slightly increased to $22,873 (₩25,777,871) per QALY gained. When applied to the different baseline distributions of NYHA class from the expert survey, the ICER greatly decreased to $3,496 (₩3,939,992) per QALY gained.

#### Scenario 2 and sensitivity analyses in the scenario 2 model

The scenario 2 model used 0.037 of utility difference according to the QoL investigated by the FAIR-HF study group. The ICER of FCM over placebo decreased to $12,598 (₩14,198,501) per QALY gained (Table 5).

When the cost ratio obtained from the study of Berry et al. [27] was applied, the ICER of FCM over placebo was $13,065 (₩14,723,924) per QALY gained. The ICERs in FCM price ± 15% ranged from $10,009 to 15,188 (₩11,279,582 to 17,117,420) per QALY gained, and the ICERs in FCM vials ± 1 vial ranged from $8,282 to $16,915 (₩9,333,636 to ₩19,063,366) per QALY gained. CHF medical cost ± 25% resulted in ICERs ranging from $11,370 to 13,827 (₩12,814,079 to ₩15,582,922) per QALY gained, which decreased as CHF medical cost increased. Delayed effect onset did not influence the results. Finally, the baseline distribution of CHF patients from the expert survey greatly decreased the ICER to $5,165 (₩5,821,178) per QALY gained.

## Discussion

This study analyzed the cost-effectiveness of FCM, an IV iron preparation, in comparison with no iron treatment in CHF patients with IDA under the Korean healthcare system. The FAIR-HF study team, Gutzwiller et al. [19], also evaluated the cost-effectiveness of FCM based on the FAIR-HF trial. However, this study had distinct differences compared with our study. First, Gutzwiller et al. evaluated cost-effectiveness from the UK National Health Service (NHS) perspective, but we analyzed cost-effectiveness from the Korean healthcare system perspective. Great differences exist between the 2 countries in terms of resource use pattern in healthcare, healthcare cost, and so on. Especially because no cost-effectiveness study has been conducted in Asia, our study is significant in that it is the first study performed under Asian healthcare system. Second, the models used differed in both outcome and cost source. Above all, the outcome in Gutzwiller et al. was based on improvement of patients’ QoL by IDA treatment, one of the main outcomes in FAIR-HF trial. The QoL was converted to utility; utility gains resulted from improved scores of QoL perceived by the study population. Meanwhile, the outcome in the base-case model of our study was based on improvement in NYHA class, another main outcome in FAIR-HF trial, which is the most commonly used outcome in cost-effectiveness studies on CHF. Thus the utility gains in our study resulted from the improvement in NYHA class. The utilities at the health status of the CHF patients with NYHA classes I, II, III, and IV were obtained from the literature search, from a QoL study targeted on the general population. Meanwhile, we also evaluated the cost-effectiveness in the scenario 2 analysis with utility gains from the FAIR-HF study. Finally, the difference in medical cost excluding IDA treatment cost in the study by Gutzwiller et al. was due to the difference in length of hospital stay between the placebo and FCM groups, whereas the difference in our study was due to the different distributions of the CHF patients with NYHA classes I, II, III, and IV because of the different improvement rates in NYHA class between the placebo and FCM groups.

In the base-case analysis, the ICER of FCM over placebo was $22,192 per QALY gained. Considering that the threshold for willingness to pay is within 1–2 times as much as the gross domestic product per capita [28], this result can be considered as acceptable in Korea. IV iron therapy with FCM is regarded as cost-effective because of the great improvement in CHF clinical status, even after taking into account the costs of the drug therapy. In addition, the results from the various sensitivity analyses were generally robust. Variations in CHF medical cost or utility weights had little effect on the results of the CEA. The FCM medication cost did affect the result relatively largely, but the ICER was still within an acceptable level. The variation in utility weight of NYHA classes II and III affected the cost-effectiveness relatively greater than the variation in utility weight of NYHA classes I and IV. This was because most patients had NYHA class II and III conditions, as shown in Table 2. Meanwhile, as the utility weights of NYHA classes I and II increased, the ICER decreased; but as the utility weights of NYHA classes III and IV increased, the ICER increased. Because FCM had a greater effect on the improvement in CHF NYHA class, the result can be considered as favorable for FCM under the condition that the difference in utility weights increases between the improved state of CHF (NYHA classes I and II) and the deteriorated state of CHF (NYHA classes III and IV). That is, the result is favorable to FCM when utilities in NYHA classes I and II are high and ones in NYHA classes III and IV are low.

This study has the following limitations: First, we conducted a systematic review to investigate clinical outcomes between FCM therapy and no iron treatment, or between FCM and other iron preparations in CHF patients with IDA. In this regard, we found only a limited number of clinical studies. Especially when we considered improvement in CHF NYHA class as the outcome, only FAIR-HF trial was useful. No study was found even for indirect comparison between FCM and other iron preparations. Thus we could not evaluate cost-effectiveness between FCM and other active comparators. In addition, the cost-effectiveness was based on the result of only one study for the same reason, even though FAIR-HF trial was a well-established, multicenter, randomized, controlled, and double-blinded study. Second, FAIR-HF trial was conducted in Europe, with a study population composed of mostly Caucasians. Therefore, the differences in baseline distribution of the NYHA classes and efficacy between Asians and Caucasians should be considered. Even though we conducted a sensitivity analysis of the baseline distribution of NYHA classes obtained from the expert survey, we were unable to apply the efficacy evaluated in Asians because no data were available. Third, no domestic utility study was available in the literature; we used the results from a survey of a United Kingdom population. Finally, the model considered the difference in NYHA classes only from the baseline to the end point, but additional information is needed to reflect the changes in NYHA class at all follow-ups.

## Conclusions

In conclusion, IDA treatment with FCM improved the clinical status of the CHF patients and demonstrated cost-effectiveness because the improvement offset the cost of the drug therapy. In addition, the sensitivity analysis was performed with various input values, and the results from the analyses were generally robust. Therefore, more-aggressive treatment options for anemia in CHF patients need to be considered.

## Abbreviations

CEA: Cost-effectiveness analysis; CHF: Chronic heart failure; ESA: Erythropoietin-stimulating agent; ESC: European Society of Cardiology; FCM: Ferric carboxymaltose; Hb: Hemoglobin; ICER: Incremental cost-effectiveness ratio; IDA: Iron-deficiency anemia; IV: Intravenous; NYHA: New York Heart Association; QALY: Quality-adjusted life year.

## Competing interests

We declare that we have no competing interest. The financial supporter did not provide any input and limitation in study design, data collection and analysis, decision to publish, and preparation of the manuscript.

## Authors’ contributions

E-A L collected the data, analyzed cost-effectiveness, and wrote the first draft of the manuscript. S-E C designed the study, reviewed the economic model, interpreted the results, and revised the manuscript. H-S S participated in the design of the economic model and helped interpret the results. H-Y L collected the data. All authors read and approved the final manuscript.
